# Characterization of the vaginal microbiota in Italian women with endometriosis: preliminary study

**DOI:** 10.1007/s00404-024-07631-x

**Published:** 2024-08-16

**Authors:** Rosa Sessa, Simone Filardo, Maria Federica Viscardi, Gabriella Brandolino, Ludovico Muzii, Marisa Di Pietro, Maria Grazia Porpora

**Affiliations:** 1 Department of Public Health and Infectious Diseases, Microbiology Section, “Sapienza” University of Rome, Rome, Italy; 2 Department of Maternal and Child Health and Urology, “Sapienza” University of Rome, Rome, Italy

**Keywords:** Endometriosis, Dysbiosis, Vaginal microbiota, Metagenomic analysis

## Abstract

**Purpose:**

This cross-sectional study aims to assess the interplay between the vaginal microbiota and endometriosis.

**Methods:**

123 consecutive Italian fertile women, aged between 20 and 40 years old, were enrolled during a routine gynecological consultation; 24 were diagnosed with endometriosis and 99 did not complain of any gynecological disease. All women underwent a vaginal swab for the evaluation of the composition and diversity of vaginal microbiota by means of 16 s rDNA metagenomic sequencing.

**Results:**

Compared to women with no gynecological disease, the vaginal microbiota in women with endometriosis showed a similar abundance of *Lactobacillus* spp.; however, a statistically significant lower abundance in the genera Pseudomonas (*p* < 0.01), Bifidobacterium (*p* < 0.05), Novispirillum (*p* < 0.0000001) and Sphingomonas (*p* < 0.0000001), and a statistically significant increase in the abundance of the genera Escherichia (*p* < 0.00001), Megasphaera (*p* < 0.00001), and Sneathia (*p* < 0.0001) were observed.

**Conclusions:**

There is a complex interplay between vaginal microbiota composition and endometriosis, showing a distinct microbial signature in the bacterial genera usually found in dysbiosis.

**Supplementary Information:**

The online version contains supplementary material available at 10.1007/s00404-024-07631-x.

## What does this study add to the clinical work

**Table:** 

There is a complex interplay between vaginal microbiota and endometriosis. Microbiota of women with endometriosis is populated by bacterial genera usually found in dysbiosis.	

## Introduction

Endometriosis is a chronic estrogen-dependent inflammatory disease affecting up to 10% of women in reproductive age, characterized by the presence of endometrial-type mucosa outside the uterus. Its presentation can be variable and includes infertility and pain symptoms, such as dysmenorrhea, dyspareunia, and acyclic chronic pelvic pain (CPP), which significantly burden the quality of life of patients [1–4]. Various theories attempt to elucidate its pathophysiology, and several factors seem to contribute to its occurrence and progression, such as inflammatory, immunological, environmental, and epigenetic factors [5–12].

The management of endometriosis is personalized and influenced by the presence and intensity of symptoms, the type and the extent of lesions, and the desire for pregnancy. Endometriosis requires long-term management, maximizing the use of medical treatment to avoid repeated surgery [13]. The hormonal treatment aims at blocking the hypothalamus–pituitary–ovarian axis, inducing amenorrhea, and reducing the progression of the disease [14]. The most common treatments include progestins and combined estro-progestins, which are effective on symptoms and considered the most suitable options for long-term therapy, also in patients with deep infiltrating endometriosis (DIE) [14–18].

In the last few years, the cervicovaginal microbiota has been suggested as a contributing factor to the pathogenesis of endometriosis [19–24]. Indeed, it is well known that a vaginal microbiota dominated by Lactobacilli plays a crucial role in women’s reproductive health, by influencing both the immune system and the homeostasis of the vaginal environment [25]. In contrast, bacterial vaginosis has been linked to the development of a chronic inflammatory state, due to the disruption of the immune system, that may compromise the integrity of the epithelial barrier, and, hence, increase the risk for migration of ectopic endometrial cells [26–31].

In this scenario, we aimed to explore the interplay between the vaginal microbiota and endometriosis; in particular, the diversity and composition of vaginal microbiota was assessed via 16 s rDNA metagenomic sequencing in an Italian cohort of women affected by endometriosis.

## Materials and methods

### Study design and sample collection

This observational cross-sectional study was performed from June 1st 2022 to December 31st 2022. Italian women of reproductive age were enrolled from the patients attending the General Gynecological outpatient consultation service of the University Hospital Policlinico Umberto I for a routine consultation. Inclusion criteria were: age between 20 and 40 years old, and a recent Papanicolau test negative for malignancy or inflammation. Exclusion criteria were pre-menarche or menopause status, diabetes, malignant diseases, urinary/genital infection in the past 6 months, bowel and/or liver disorders, current treatment with prokinetics, antacids or proton pump inhibitors, sexual activity in the week prior to sampling, recent or current antibiotic treatment (oral or topical), as well as the use of probiotics and/or prebiotics at least for 3 months prior to the enrolment.

Age, body mass index (BMI), parity, comorbidities, previous surgery, use of nonsteroidal anti-inflammatory drugs (NSAIDs), estro-progestins, progestins, or other medications, presence of infertility or pain symptoms (dysmenorrhea, dyspareunia, and acyclic pelvic pain (CPP)) were recorded.

All women underwent a gynecological examination and a transvaginal ultrasound (TVUS) performed by the same operator to exclude or diagnose endometriosis (GE Voluson E6, suprapubic 3.5 MHz volume probe and transvaginal 6 MHz volume probe, with 3D scan, GE Healthcare, Milwaukee, WI, USA).

From each woman, a vaginal swab for metagenomic analysis was collected; in those who were not taking hormonal therapy, the vaginal sampling was made at the time of ovulation, as detected by the ovulation test kit “Clearblue digital test” kit (Swiss Precision Diagnostics GmbH, Geneva, Switzerland), while in women taking hormonal therapy, it was collected during gynecological consultation. All women were asked to avoid sexual intercourse in the 7 days before the sample collection. Samples were immediately stored at − 20 °C until further processing.

All study participants gave written informed consent to the study. The study was approved by the Umberto I University Hospital Ethics Committee (reference number 5930/20) and conducted according to the principles expressed in the Declaration of Helsinki.

### Metagenomic analysis

Extraction, quantification, and integrity of total genomic DNA from vaginal swabs, as well as 16 s rRNA (V3–V4 hypervariable region) gene amplification and Illumina MiSeq sequencing, were carried on as previously described [32] (Filardo et al., 2022). Bioinformatic processing of raw reads and subsequent statistical analysis (alpha and beta diversity comparisons, ANCOM, and LEfSe analysis) were performed in QIIME 2 [33].

### Statistical analysis

Parametric data, expressed as mean ± standard deviation (SD), were analyzed by Student’s *t*-test; the comparison between the groups was carried out by Fisher’s test. Relative abundances of taxa were expressed as means ± standard error of means (SEM), whereas alpha diversity indexes as median (IQR). Non-parametric *t*-test based on Monte Carlo permutations was used for alpha diversity comparisons, and Adonis was used for category comparisons of distance matrices, all calculated in QIIME 2. The single or multiple inference significance level was set at 5%.

## Results

A total of 123 consecutive women were enrolled in the study: 24 (19.5%) were diagnosed with endometriosis (Group A), amongst them, 10 were treated with dienogest 2 mg/daily for at least 6 months (Group A1), and 14 did not take any hormonal therapy (Group A2); 99 did not show any gynecological disease (Group B). The main characteristics of the study population are reported in Tables 1, 2.

**Table 1:** Characteristics of the study population

	Group A (*n* = 24)	Group B (*n* = 99)	*p* values
Age (mean ± s.d.)	27.4 ± 3.2	25 ± 5.7	n.s
BMI (mean ± s.d.)	22.5 ± 3.3	22.7 ± 4.5	n.s
Age at menarche (mean ± s.d.)	12.0 ± 1.1	12.1 ± 1.5	n.s
Regular bowel *n* (%)	24 (100)	99 (100)	n.s
Stypsis *n* (%)	0 (0)	0 (0)	n.s
Diarrhea *n* (%)	0 (0)	0 (0)	n.s
Regular diuresis *n* (%)	24 (100)	99 (100)	n.s
Recurrent cystitis *n* (%)	0 (0)	0 (0)	n.s
Smoke *n* (%)	7 (29.1)	20 (20.2)	n.s
Dysmenorrhea *n* (%)	14 (58.3)	0 (0)	0.001
Dysmenorrhea VAS (mean ± s.d.)	6.5 ± 2.2	NA	NA
Dyspareunia *n* (%)	19 (79.1)	0 (0)	< 0.001
Dyspareunia VAS (mean ± s.d.)	5.8 ± 2.1	NA	NA
CPP *n* (%)	13 (54.1)	0 (0)	< 0.001
CPP VAS (mean ± s.d.)	3.4 ± 2.4	NA	NA
NSAIDs intake *n* (%)	20 (83.3)	30 (31.25)	< 0.001
Ovarian endometriomas *n* (%)	24 (100)	NA	NA
Size of ovarian endometriomas (mean ± s.d.)	25 mm ± 18 mm	NA	NA
DIE *n* (%)	5 (20.8)	NA	NA
Size of DIE (mean ± s.d.)	11 mm ± 0.4 mm	NA	NA
Previous surgery for endometriosis *n* (%)	8 (33.3)	NA	NA

Group A, women with endometriosis; Group B, women with no gynecological disease

*n.s* not significant, *NA* not applicable, *s.d* standard deviation, *VAS* visual analog scale

**Table 2:** Characteristics of the study population according to the hormonal therapy

	Group A1 (*n* = 10)	Group A2 (*n* = 14)	*p* values
Age (mean ± s.d.)	27.0 ± 2.7	28.6 ± 3.1	n.s
BMI (mean ± s.d.)	22.1 ± 3.8	23.2 ± 3.4	n.s
Age at menarche (mean ± s.d.)	12.0 ± 1.2	11.9 ± 1.1	n.s
Regular bowel *n* (%)	5 (50)	7 (50)	n.s
Regular diuresis *n* (%)	9 (90)	12 (85.7)	n.s
Recurrent cystitis *n* (%)	0 (0)	0 (0)	n.s
Smoke *n* (%)	4 (40)	3 (21.4)	n.s
Dysmenorrhea *n* (%)	NA	14 (100)	NA
Dysmenorrhea VAS (mean ± s.d.)	NA	6.5 ± 2.2	NA
Dyspareunia *n* (%)	8 (80)	11 (78.5)	n.s
Dyspareunia VAS (mean ± s.d.)	1.2 ± 2.5	6.2 ± 1.6	0.001
CPP *n* (%)	5 (50)	8 (57.1)	n.s
CPP VAS (mean ± s.d.)	1.4 ± 1.7	5.8 ± 1.6	0.001
NSAIDs intake *n* (%)	6 (60)	14 (100)	0.01
Ovarian endometriomas *n* (%)	10 (100)	14 (100)	n.s
Size of ovarian endometriomas (mean ± s.d.)	25 mm ± 18 mm	23 mm ± 16 mm	n.s
DIE *n* (%)	2 (20)	3 (21.4)	n.s
Size of DIE (mean ± s.d.)	11 mm ± 0.4 mm	10.6 mm ± 0.3 mm	n.s
Previous surgery for endometriosis *n* (%)	3 (30)	5 (35.7)	n.s

Group A1, women with endometriosis taking dienogest; group A2, women with endometriosis and no hormonal therapy

*n.s* not significant, *NA* not applicable, *s.d* standard deviation, *VAS* visual analog scale

Group A and group B were well matched for the several clinical factors examined, except for dysmenorrhea (*p* = 0.001), dyspareunia *(p* < 0.001*),* CPP *(p* < 0.001*),* and NSAIDs intake (*p* < 0.001), which were significantly more frequent in group A.

### Composition of vaginal microbiota in the study population

An average of 27,340 [median (Interquartile Range, IQR) 20,871 (11,492.25)] and 72,001 [79530 (32,535)] paired-end Illumina reads were analyzed in vaginal swabs from women with endometriosis and women with no gynecological disease, respectively, by metagenomic analysis of the hypervariable region V3–4 from the bacterial 16 s rDNA via Illumina sequencing. After the removal of singletons and rare Operational Taxonomic Units (OTUs), a total number of 57 [9.5 (5.75)] and 49 [14 (7)] OTUs were identified in women with endometriosis and women with no gynecological disease, respectively. The lowest read was 3714 and, hence, the OTUs were randomly subsampled to this minimum read for diversity analysis to avoid bias. There were no statistically significant differences in the number of OTUs between women with endometriosis and women with no gynecological pathology, showing similar sequencing results.

First, the vaginal microbiota composition in all women with endometriosis was compared to women with no gynecological disease, as shown in Table 3 and supplementary Fig. 1A. The vaginal microbiota in women with endometriosis showed a statistically significant decrease in the relative abundance of the genera *Pseudomonas* (*p* < 0.01), *Bifidobacterium* (*p* < 0.05), *Novispirillum* (*p* < 0.0000001) and *Sphingomonas* (*p* < 0.0000001), typically associated to a healthy vaginal microbiota, whereas a statistically significant increase could be observed in the relative abundance of the genera *Escherichia* (*p* < 0.00001), *Megasphaera* (*p* < 0.00001), and *Sneathia* (*p* < 0.0001), as compared to the vaginal microbiota in women with no gynecological pathology.

**Table 3:** Vaginal microbiota composition in the study population at the genus level, in relation to the presence of endometriosis

	Group A (%) (*n* = 24)	Group B (%) (*n* = 99)	*p* values
*Lactobacillus*	88.48	85.85	N.S
*Gardnerella*	3.92	6.03	N.S
*Pseudomonas*	0.02	1.89	0.007
*Bifidobacterium*	0.02	1.48	0.031
*Fannyhessea*	0.64	1.34	N.S
*Novispirillum*	0.01	0.83	< 0.0000001
*Limosilactobacillus*	0.32	0.78	N.S
*Lacticaseibacillus*	0.00	0.32	N.S
*Prevotella*	1.02	0.25	N.S
*Sphingomonas*	0.00	0.24	< 0.0000001
*Streptococcus*	0.12	0.23	N.S
*Staphylococcus*	0.11	0.11	N.S
*Escherichia*	2.26	0.03	0.000002
*Megasphaera*	0.90	0.00	0.000006
*Sneathia*	0.68	0.00	0.00008
Others	1.51	0.63	N.S

Group A, women with endometriosis; Group B, women with no gynecological diseases

*N.S* not significant

Second, to assess the possible influence of oral progestins on the vaginal microbiota composition of women with endometriosis, we compared women taking dienogest with women without any hormonal therapy. As evidenced in Table 4 and Supplementary Fig. 1B, the vaginal microbiota composition in these two groups is very similar; however, women with endometriosis who did not take any hormonal therapy showed a slight increase in the genera *Gardnerella*, *Prevotella*, *Megasphaera*, and *Sneathia*, alongside a decrease in the genus *Escherichia*, although these results did not reach statistical significance.

**Table 4:** Vaginal microbiota composition in the study population at the genus level, in relation to the hormonal treatment

	Group A1 (%) (*n* = 10)	Group A2 (%) (*n* = 14)	*p* values
*Lactobacillus*	91.63	87.10	N.S
*Gardnerella*	0.40	5.45	N.S
*Pseudomonas*	0.04	0.01	N.S
*Bifidobacterium*	0.07	0.00	N.S
*Fannyhessea*	0.09	0.88	N.S
*Novispirillum*	0.00	0.00	N.S
*Limosilactobacillus*	0.44	0.27	N.S
*Lacticaseibacillus*	0.00	0.00	N.S
*Prevotella*	0.10	1.42	N.S
*Sphingomonas*	0.00	0.00	N.S
*Streptococcus*	0.35	0.01	N.S
*Staphylococcus*	0.20	0.07	N.S
*Escherichia*	4.64	1.23	N.S
*Megasphaera*	0.07	1.26	N.S
*Sneathia*	0.02	0.97	N.S
Others	1.94	1.32	N.S

Group A1, women with endometriosis taking dienogest; Group A2, women with endometriosis and no hormonal therapy

*N.S* not significant

### Alpha- and *beta*-diversities analysis

**Fig. 1:**
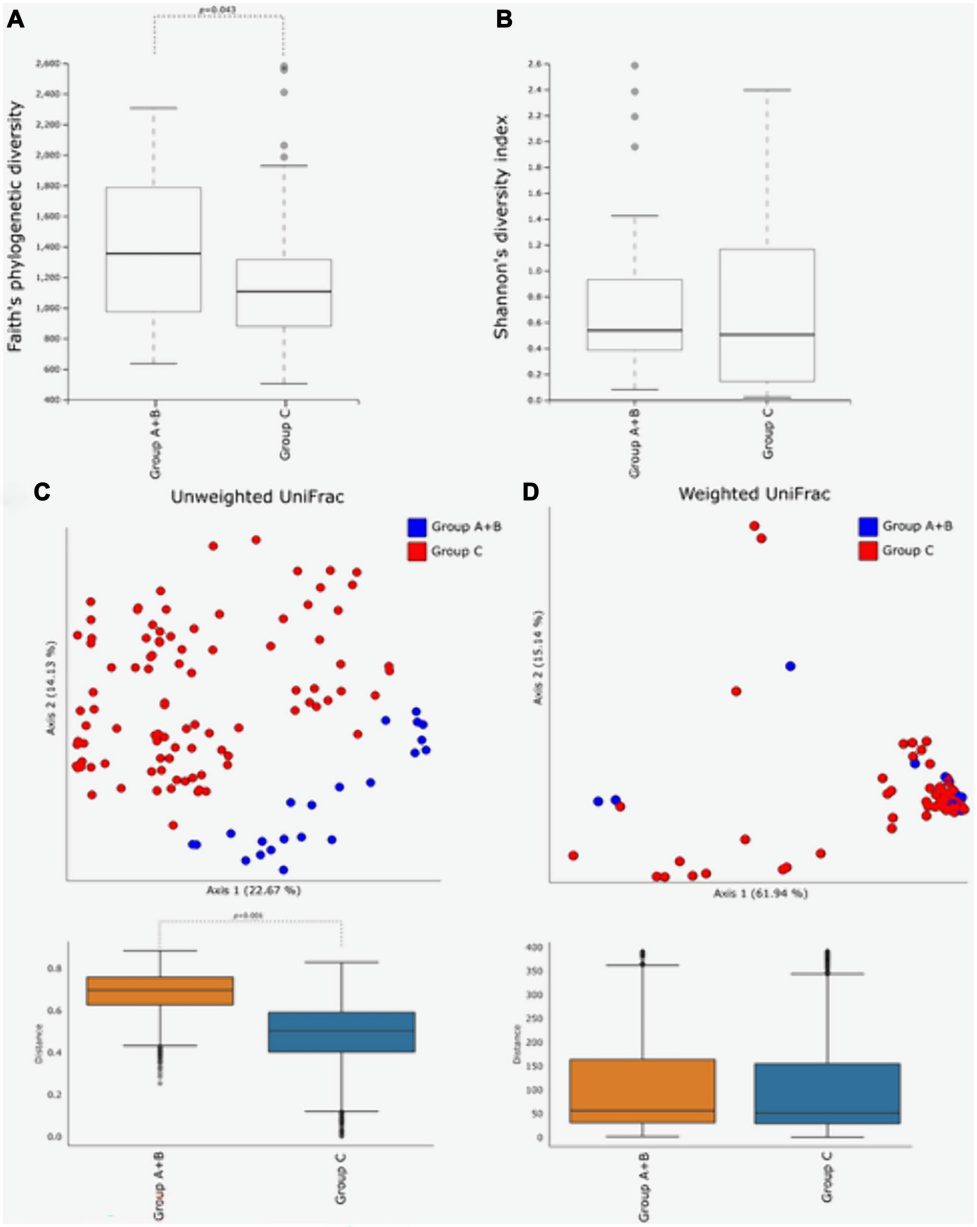
Comparison of the alpha- and beta-diversity of the vaginal microbiota in relation to the presence of endometriosis. Faith’s phylogenetic diversity (**A**) and Shannon’s diversity index (**B**) were used to measure alpha-diversity within groups. The circles out of range represent the outliers. Principal coordinate analysis (PCoA) plots, and boxplot representations of within-group distances, of unweighted (**C**) and weighted (**D**) UniFrac distance matrices, are illustrated. Each dot represents the vaginal bacterial community composition of one individual. Groups were compared using Adonis for beta-diversity. F. Samples were rarefied to the smallest observed number of reads (3714). Group A, all women with endometriosis; group B, women with no gynecological disease

Comparing the vaginal microbiota between women with endometriosis and women with no gynecological disease, the Faith’s phylogenetic diversity showed a significantly higher diversity in the presence of endometriosis (Fig. 1A, *p* < 0.05). In contrast, the Shannon’s diversity index did not show any statistically significant difference between the two groups (Fig. 1B). Concerning the beta-diversity measures, a statistically significant clustering of bacterial communities from the vaginal microbiota of women with endometriosis as compared to women with no gynecological disease was evidenced in the unweighted (*p* < 0.001) UniFrac analysis, whereas the weighted UniFrac analysis did not evidence any statistically significant clustering (Fig. 1C, D).

Subsequently, we investigated whether there were differences in the diversity and richness of the bacterial communities found in the vaginal micro-environment of women with endometriosis in relation to treatment with dienogest, via Faith’s phylogenetic diversity and Shannon’s diversity index, as measures of alpha-diversity, and the weighted and unweighted UniFrac distance matrices, as measures of beta- diversity. No difference in either Faith’s phylogenetic diversity or Shannon’s diversity index was observed in relation to hormonal treatment (supplementary Fig. 2A, B); similarly, no statistically significant clustering was observed by either the unweighted or weighted UniFrac analysis (supplementary Fig. 2C, D).

### Specific taxonomic units as potential biomarkers

To identify specific taxa as potential biomarkers associated with endometriosis condition or control group, two different approaches, namely the linear discriminant analysis (LDA) coupled with effect size measurement (LEfSe), and the Analysis of Composition of Microbiomes (ANCOM), were used.

**Fig. 2:**
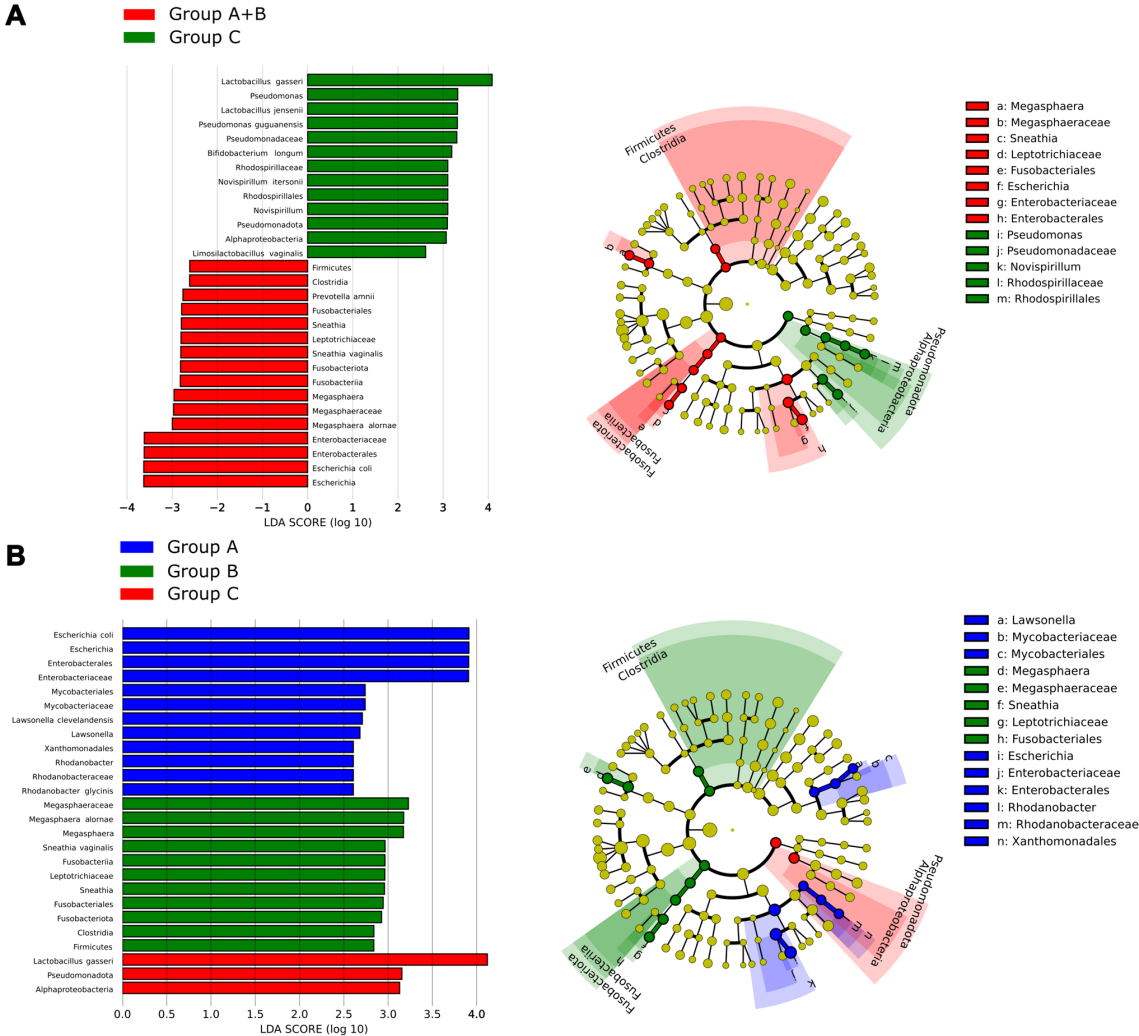
Linear discriminant analysis with effect size measurement (LEfSe) of the vaginal microbiota in relation to the presence of endometriosis (**A**), and in relation to the hormonal therapy (**B**). On the left, histograms of the LDA scores were computed for statistically significant differentially abundant taxonomic units between the groups. On the right, cladograms highlight the relationships of the significantly different taxonomic units between the groups. Differences are represented in the color of the most abundant class, and each circle’s diameter is proportional to the taxon’s abundance. Group A, all women with endometriosis; Group B, women with no gynecological condition; group A1, women with endometriosis taking dienogest; Group A2, women with endometriosis and no hormonal therapy

In particular, the LEfSe analysis highlighted a statistically significant association of the genera *Lactobacillus* spp. (specifically *L. gasseri* and *L. jensenii*, LDA > 3.0), *Pseudomonas* spp. (specifically *Pseudomonas guguanensis*, LDA > 3.0) and *Bifidobacterium* spp. (specifically *Bifidobacterium longum*, LDA > 3.0) with women with no gynecological disease, whereas the genera Prevotella spp. (specifically *Prevotella amnii*, LDA > 2.5), *Sneathia* spp. (specifically *S. vaginalis*, LDA > 2.5), *Megasphaera* spp. (specifically *Megasphaera alornae*, LDA > 2.5), and *Escherichia* spp. (specifically *Escherichia coli*, LDA > 3.0), were significantly associated with the vaginal microbiota found in patients with endometriosis (Fig. 2A). Interestingly, patients with endometriosis who did not take dienogest were significantly related to the presence of *Megasphaera* spp. and *Sneathia* spp. (LDA > 2.5), whereas patients with endometriosis who did take dienogest had a stronger association with *Escherichia* spp. (LDA > 3.5), as well as *Mycobacteriaceae* (LDA > 2.5), *Rhodanobacteraceae* (LDA > 2.5), and *Enterobacteriaceae* (LDA > 3.5) (Fig. 2B).

**Fig. 3:**
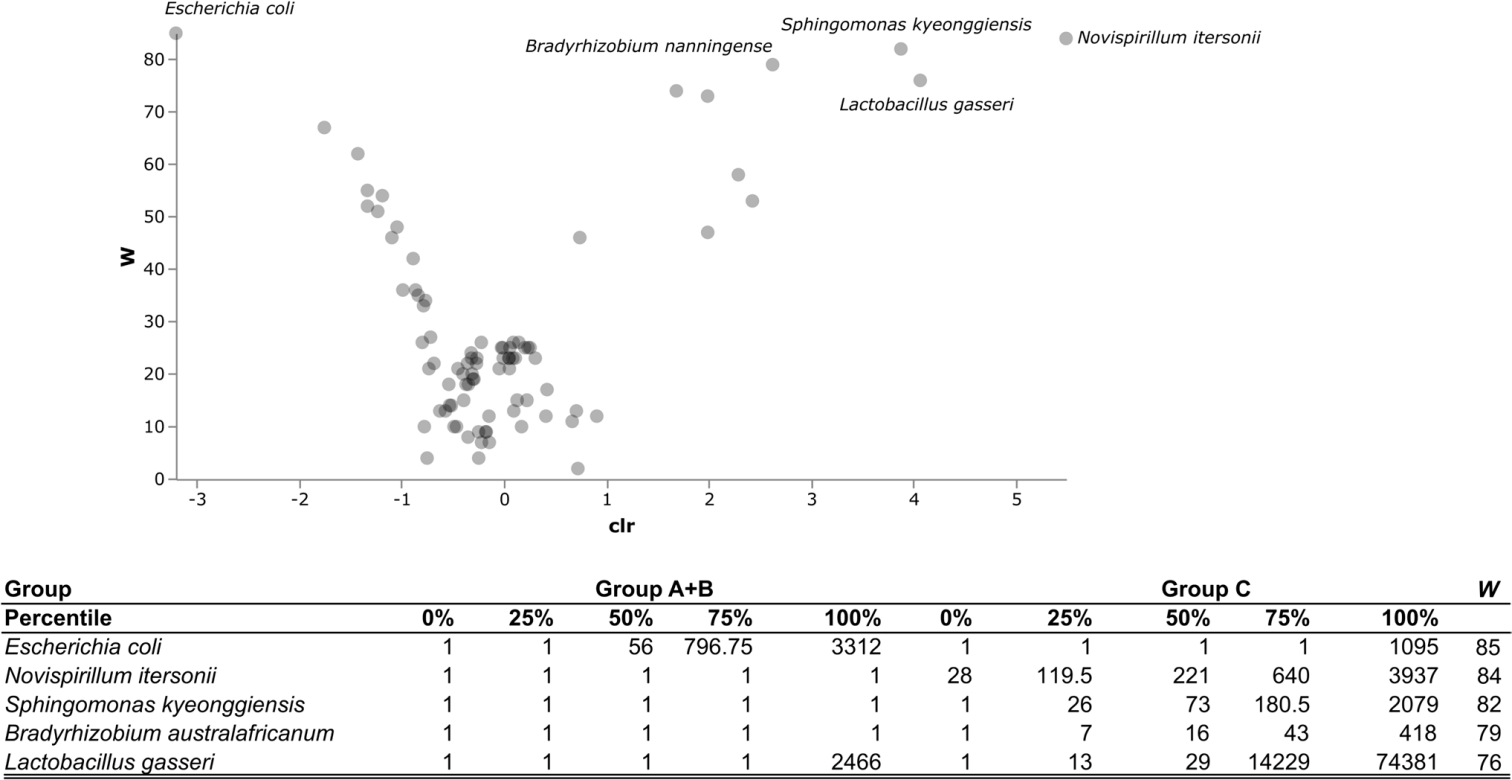
ANCOM test of the vaginal microbiota between endometriosis patients and women with no gynecological disease. *W* statistics represent the number of times the null hypothesis is rejected for a given taxon. Group A, all women with endometriosis; group B, women without gynecological diseases

**Fig. 4:**
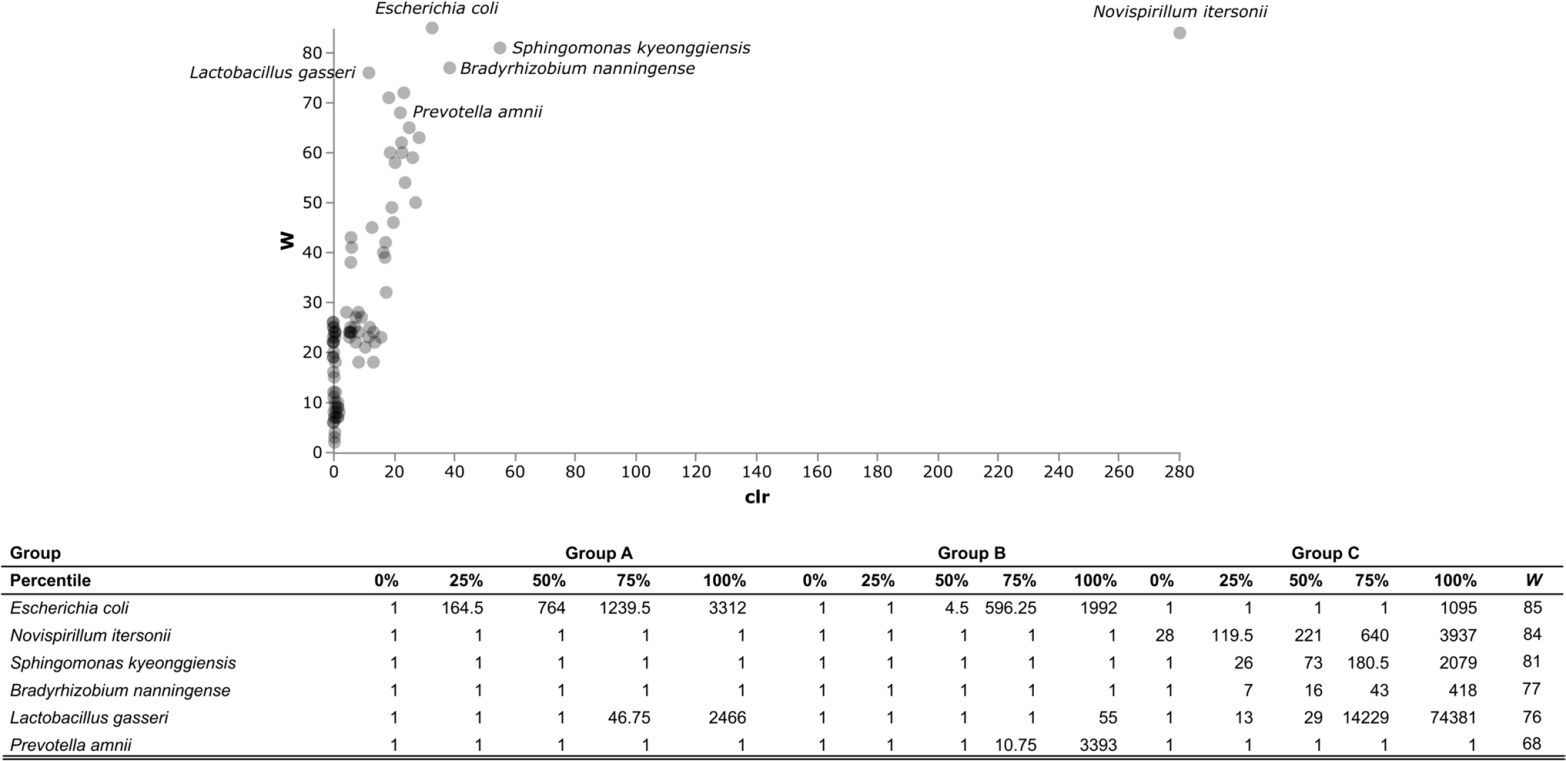
ANCOM test of the vaginal microbiota amongst women with endometriosis in relation to the hormonal therapy, and women without any gynecological disease. *W* statistics represent the number of times the null hypothesis is rejected for a given taxon. Group A1, women with endometriosis taking dienogest; group A2, women with endometriosis and no hormonal therapy; group B, women with no gynecological disease

The ANCOM test confirmed the statistically significant association of *L. gasseri* with the absence of gynecologic conditions, while *E. coli* resulted strongly associated with endometriosis patients. Moreover, *Novispirillum itersonii*, *Sphingomonas kyeonggiensis,* and *Bradyrhizobium australafricanum* were also significantly associated to the absence of gynecological conditions, as evidenced in Fig. 3. Similarly, *E. coli* was more prevalent in women with endometriosis treated with dienogest, whereas those without any hormonal treatment had a significant association with *P. amnii* (Fig. 4).

## Discussion

Changes in vaginal microbiota of patients with endometriosis have been only recently investigated, but few conflicting data, resulting from studies conducted on non-homogeneous populations, differing in ethnic characteristics and dietary habits, are available [24]. Most studies have focused on the effect of hormones on the gut microbiota, comparing the composition in postmenopause and in the reproductive age; however, the results are sparse and inconclusive [34]. There is no data on the influence that hormonal therapy has on the vaginal microenvironment of endometriosis patients, although they often take hormones chronically.

Our study aimed to identify differences in the diversity and richness of vaginal microbiota in relation to the presence of endometriosis. Overall, women with endometriosis and women with no gynecological disease possessed a *Lactobacillus*-dominated vaginal microbiota, suggesting a baseline concordance in their microbial communities. However, a significantly higher bacterial diversity, as indicated by Faith’s phylogenetic diversity, was associated with endometriosis. In particular, statistically significant differences have emerged in the relative abundance of the less represented bacterial genera, such as a decrease in *Pseudomonas*, *Bifidobacterium*, *Novispirillum*, and *Sphingomonas*, alongside an increase in *Escherichia*, *Megasphaera*, and *Sneathia* in women with endometriosis, suggesting a distinct microbial signature, albeit it could not be defined as vaginal dysbiosis due to the prevalence of *Lactobacillus* spp., as also evidenced by other studies [23].

Interestingly, the higher abundance of *Escherichia coli* in the vaginal microbiota from women with endometriosis as compared to women with no gynecological disease (*p* < 0.01), suggested its potential involvement in the pathogenesis of endometriosis. Further, supporting our findings, Ata et al. have evidenced an increase in *Escherichia* spp. in the cervicovaginal microbiota of women with endometriosis as compared to healthy controls [22]; this evidence, alongside our findings, opens a novel scenario in the pathophysiology of endometriosis. *Escherichia* spp. is not a bacterial-vaginosis (BV)-associated microorganism but can be considered an opportunistic pathogen in the cervicovaginal microbiota that contributes to endometriosis by inducing inflammation. Indeed, there is evidence in the literature that the menstrual blood of patients with endometriosis is more contaminated by *E. coli*, with higher levels of endotoxin, than that of healthy patients, suggesting that the menstrual blood reflux in the peritoneal cavity could trigger natural immunity by activation of TRL-4, leading to chronic inflammation and, hence, contributing to the development of endometriosis [35]. However, an *E. coli* transient colonization of the vaginal microenvironment cannot be excluded, albeit this hypothesis is rather unlikely due to the strict inclusion criteria adopted for the enrollment of our population, including the absence of sexual intercourse for at least a week prior to sampling, the recommendation over personal hygiene practices, and exclusion of signs and symptoms of urinary tract infections.

Given that women with endometriosis often chronically take hormones, like progestins, to reduce the progression of the disease and to treat their symptoms, it has also been interesting to investigate the influence that hormonal therapy might have on their vaginal microenvironment. In our study, endometriosis patients taking dienogest had a lower abundance of bacterial species classically associated with dysbiosis, albeit not statistically significant, including *Gardnerella* spp., *Prevotella* spp., *Megasphaera* spp., and *Sneathia* spp., and a higher abundance of *E. coli*, as compared to women with endometriosis and no hormonal therapy; this scenario could be due to the anti-inflammatory effect of dienogest [36–38].

The higher abundance of *E. coli* observed in the patients receiving dienogest as compared to those with no hormonal therapy, albeit not statistically significant, underlines the complex multifactorial etiopathogenesis of endometriosis, suggesting the presence of a dynamic balance between pro- and anti-inflammatory factors.

A limitation of our study was the small sample size of the patient groups according to the hormonal treatment; however, the results are interesting, and further studies will be necessary to reveal the potential role of *Escherichia coli* in the pathogenesis of endometriosis and its link with the hormonal treatment.

In conclusion, this study may add a piece to the puzzle for understanding the complex interplay of the vaginal microbiota composition and endometriosis, showing a peculiar microbial signature in women with endometriosis. Future research employing large randomized longitudinal studies and functional metagenomic approaches will help provide a more comprehensive understanding of this relationship.

## Supplementary Information

Below is the link to the electronic supplementary material.

Supplementary file1 Vaginal microbiota composition in the study population in relation to endometriosis (**A**), or to the hormonal treatment (**B**). Only taxa with abundances greater than 0.01% in any sample were included in the graphs. Group A, all women with endometriosis; Group B, women with no gynecological pathological condition; Group A1, women with endometriosis taking dienogest; Group A2, women with endometriosis and no hormonal therapy (TIFF 810 KB)

Supplementary file2 Comparison of the alpha- and beta-diversity of the vaginal microbiota in relation to the hormonal therapy in women with endometriosis. Faith’s phylogenetic diversity (**A**) and Shannon’s diversity index (**B**) were used to measure alpha-diversity within groups. The circles out of range represent the outliers. Principal coordinate analysis (PcoA) plots, and boxplot representations of within-group distances, of unweighted (**C**) and weighted (**D**) UniFrac distance matrices, are illustrated. Each dot represents the vaginal bacterial community composition of one individual. Groups were compared using Adonis for beta-diversity. F. Samples were rarefied to the smallest observed number of reads (3714). Group A1, women with endometriosis taking dienogest; group A2, women with endometriosis and no hormonal therapy (TIFF 894 KB)

## Data Availability

Data will be provided by the authors on request.
