# Supplementary figures and images for: Characterization of the vaginal microbiota in Italian women with endometriosis: preliminary study
Source: Arch Gynecol Obstet. 2024 Aug 16;310(4):2141–51. doi: 10.1007/s00404-024-07631-x (PMC11393154; doi:10.1007/s00404-024-07631-x)

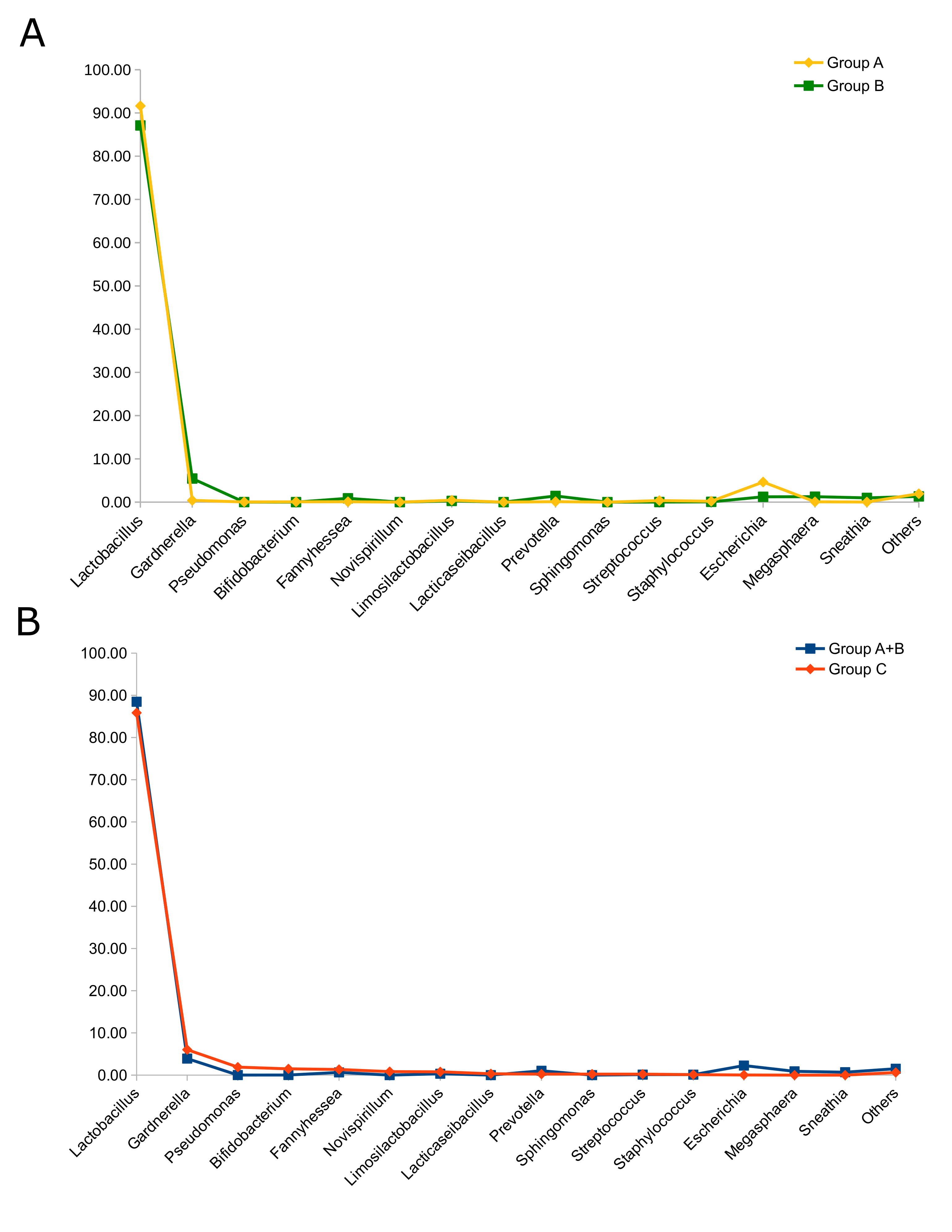

Supplement: Supplementary file 1 — Supplementary file1 Vaginal microbiota composition in the study population in relation to endometriosis (A), or to the hormonal treatment (B). Only taxa with abundances greater than 0.01% in any sample were included in the graphs. Group A, all women with endometriosis; Group B, women with no gynecological pathological condition; Group A1, women with endometriosis taking dienogest; Group A2, women with endometriosis and no hormonal therapy (TIFF 810 KB) [file 404_2024_7631_MOESM1_ESM.tiff]

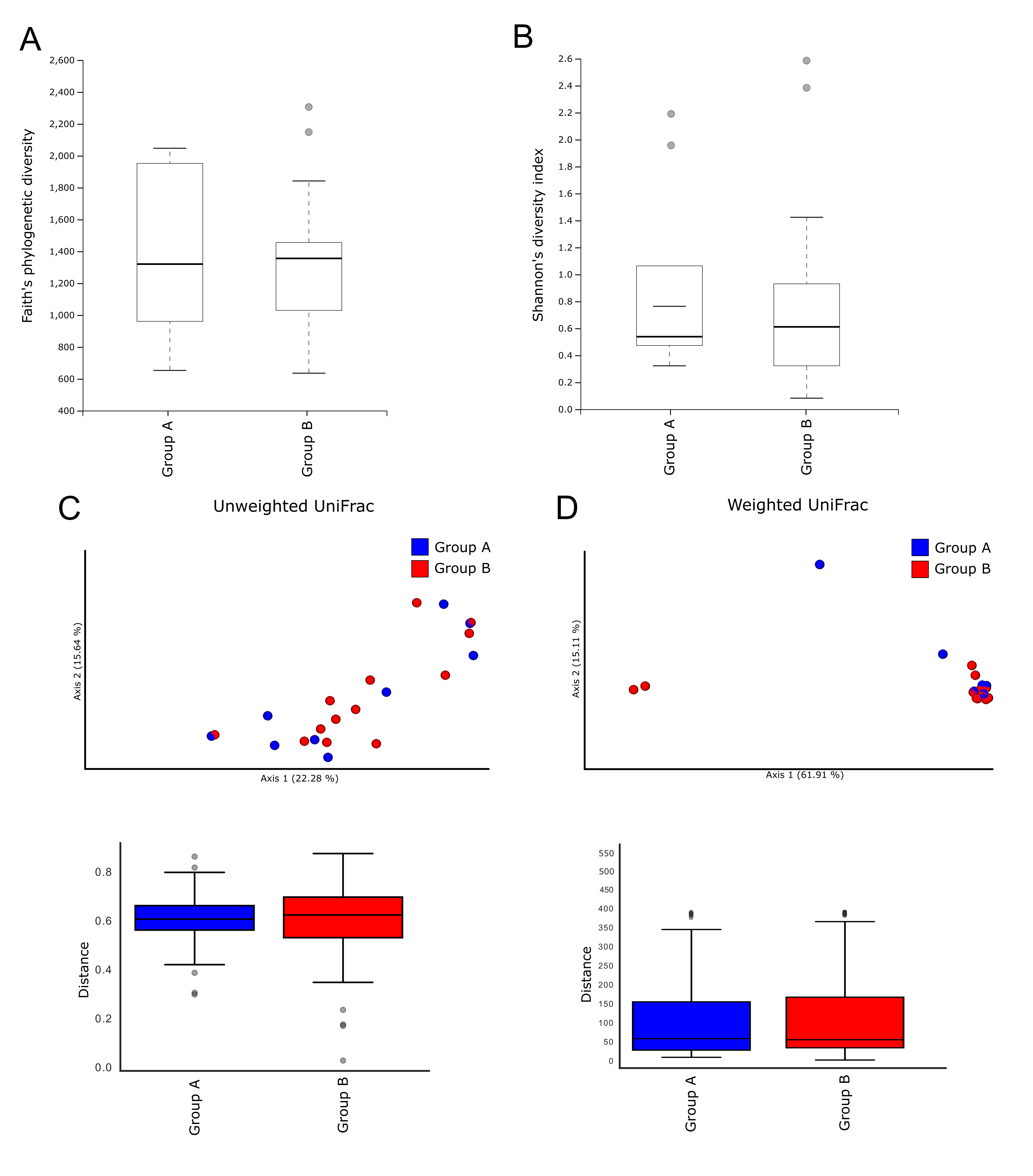

Supplement: Supplementary file 2 — Supplementary file2 Comparison of the alpha- and beta-diversity of the vaginal microbiota in relation to the hormonal therapy in women with endometriosis. Faith’s phylogenetic diversity (A) and Shannon’s diversity index (B) were used to measure alpha-diversity within groups. The circles out of range represent the outliers. Principal coordinate analysis (PcoA) plots, and boxplot representations of within-group distances, of unweighted (C) and weighted (D) UniFrac distance matrices, are illustrated. Each dot represents the vaginal bacterial community composition of one individual. Groups were compared using Adonis for beta-diversity. F. Samples were rarefied to the smallest observed number of reads (3714). Group A1, women with endometriosis taking dienogest; group A2, women with endometriosis and no hormonal therapy (TIFF 894 KB) [file 404_2024_7631_MOESM2_ESM.tiff]
